# Estimation of Probable Maximum Precipitation in the context of climate change

**DOI:** 10.1016/j.mex.2020.100904

**Published:** 2020-04-28

**Authors:** Subharthi Sarkar, Rajib Maity

**Affiliations:** Indian Institute of Technology Kharagpur, India

**Keywords:** Annual maximum daily precipitation, Enveloping technique, Frequency factor

## Abstract

Probable Maximum Precipitation (PMP) is the maximum depth of precipitation at a location for a given duration that is meteorologically possible. It is a crucial information for any water infrastructure, such as dams, culverts, drainage network in order to ensure a desirable probability of exceedance. This paper proposes a technique for estimation of PMP, suitable in the context of climate change. Out of several available methods, Hershfield method is considered as a convenient and effective statistical method of PMP estimation, provided sufficiently long precipitation records are available. The most crucial step in Hershfield method is the precise estimation of frequency factor (*K*) and its enveloping technique. There is no universally accepted enveloping technique of *K*. Different values of *K* and different types of enveloping techniques have been suggested and used by various investigators across the world. We introduce an upgradation in the existing enveloping technique in order to bring clarity and universality in the estimation, particularly in the context of climate change. This updated enveloping technique and the conventional Hershfield method-both are applied to develop PMP maps for the entire Indian mainland over the past century (1901-2000). Comparison between the proposed and existing methods of PMP estimation reveals a better estimation of spatio-temporal variation of PMP, avoiding unusual overestimation of PMP in the low rainfall extreme regions of India by existing Hershfield method.

In brief, the contributions of this paper are as follows:

• An upgradation of the existing Hershfield Method [1] by introducing a new enveloping technique for the frequency factor (*K*).
• The single envelope curve in the existing Hershfield method is modified as a composite curve, consisting of a straight-line portion and an exponentially decaying portion.
• Development of PMP maps over India using both Hershfield method and the proposed technique

## Graphical abstract

**Figure:**
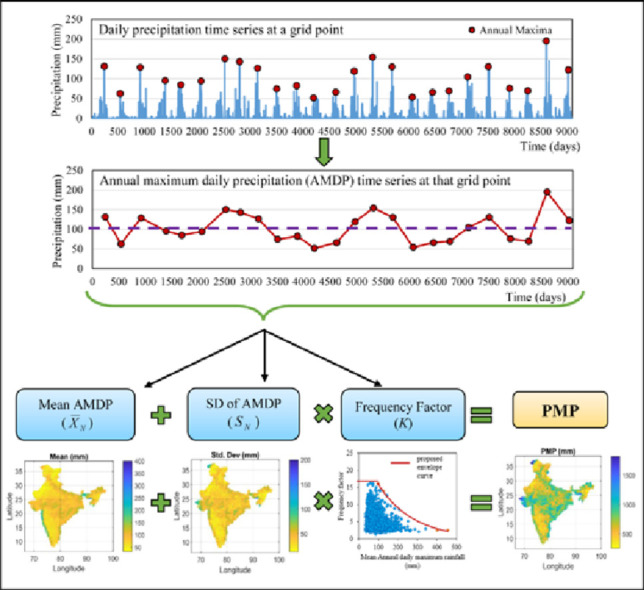

**Table:** Specifications Table

Subject Area:	Earth and Planetary Sciences
More specific subject area:	Hydroclimatology
Method name:	Statistical reconstruction method for PMP estimation
Name and reference of original method:	Hershfield method (1965) for PMP estimation. Reference: D.M. Hershfield, Method for estimating probable maximum precipitation, J. Am. Waterworks Assoc. 57 (1965) 965–972.
Resource availability:	S. Sarkar, R. Maity, High-resolution One-day Probable Maximum Precipitation dataset across India and its Future-projected Changes over India, Mendeley Data, v1, http://dx.doi.org/10.17632/zx99xr4wkr.1[2]

## Introduction

Probable Maximum Precipitation (PMP) is ‘the greatest depth of precipitation for a given duration meteorologically possible for a design watershed or a given storm area at a particular location at a particular time of year, with no allowance made for long-term climatic trends’ [3]. It is an important hydrologic information for the estimation of Probable Maximum Flood (PMF) [4], which is used in the design of many major, medium and minor hydraulic structures such as large dams [5], high-risk energy infrastructures like nuclear power plants [6], weirs and barrages, culverts and cross-drainage works.

However under this present scenario of changing climate, similar to various other hydroclimatic variables, PMP is also expected to change over the years. The global warming and consequent intensification of hydrological cycle acts as a major driving force behind such changes. Several recent studies at global and regional scale have investigated on the possible impact of climate change on PMP, and reported about a general increasing trend in different parts of the world. For instance, [7] showed that the PMP is expected to increase in future all over the world due to substantial increase in atmospheric moisture content and consequent higher levels of moisture transport into storms. [8] and [9] detected a tendency of increasing PMP/PMF estimates over different watersheds in Quebec, Canada after analyzing different downscaled climate model projections. [10] and [11] also projected a significant increase in PMP/PMF estimates (20% and 44% increase in PMP in the 2021–2050 and 2071–2100 time periods, respectively w.r.t 1981–2010 baseline values) over the Alabama-Coosa-Tallapoosa (ACT) River Basin in the south-eastern United States. [12] reported an increase in PMP by 50% ± 30% around 2099 w.r.t. 2016 level, in the Pacific Northwest (PNW) region in the USA. [13] projected around 30% increase in PMP under RCP 8.5 scenario in South Korea. [14] estimated PMP to increase up to 18.2% and 27.3% in Southern Iran across different GCMs toward the end of the century w.r.t. 1971-2000 base period. Likewise, several other studies have also projected different extents of increase in PMP in different parts of the world, e.g., South Korea [15], Vietnam [16], India [17], Iran [18] and many more. Such an extent of likely changes in PMP is expected to have some serious implications on those afore-mentioned major water-energy structures in future due to their very long life span (>100–500 years) by altering their design risk and reliability over the years [7,19]. Thus, Proper estimation of PMP in the context of climate change is very important considering the devastations upon failure of those high-risk structures.

Various methods are available in literature (e.g., [1,[20], [21], [22], [23], [24], [25]]) for estimation of PMP, which can be broadly classified into two categories, namely, physical methods and statistical methods. Among them, Hershfield method [1,25] is considered as a convenient, popular and efficient statistical tool for the estimation of PMP. This method is particularly useful for those locations where sufficiently long precipitation records are available but other meteorological data (e.g., dew point temperature, wind speed, relative and humidity) are lacking, which are essential requirements for other physical methods, such as moisture maximization and storm transposition technique [5,26]. With the availability of sufficiently long record of precipitation, the PMP estimates by Hershfield method are found to be closely comparable to those by other physical approaches at different parts of the world, e.g., [27] for Canada, [28] for the USA, [29] for Iceland and [20] for Australia. The World Meteorological Organization (WMO) has also recommended the Hershfield method as one of the methods to estimate PMP in their various manuals and technical publications [5].

In Hershfield method of PMP estimation, only the Annual Maximum Daily Precipitation (AMDP) series is required for a particular location, from which the frequency factor (*K*) is estimated. In general, frequency factor (*K*) can be defined as the standardized version of the residual maximum rainfall (i.e., maximum AMDP minus mean AMDP) [1,30]. The standardization is done by dividing the residual maximum rainfall by the standard deviation of AMDP series. Thus the frequency factor is analogous to the standard normal variate or reduced variate in case of analysis of normally distributed data or extreme-value series, respectively. However, the most vital step in this method of PMP estimation is the enveloping of this frequency factor (*K*). Lack of a universally accepted enveloping technique of *K* has lead various researchers across the world to use different types of enveloping techniques [5]. However, the conventional method of single exponential upper envelope curve for frequency factor (*K*), as proposed by Hershfield [1] provides unrealistic weightage to the comparatively dry regions with very low values of mean AMDP magnitude. To overcome that, the present study splits this exponentially decaying curve into two parts, viz., one straight-line part having *K* value equal to the maximum value of *K* in the region of interest, and the remaining portion follows the conventional exponential curve. Further details on the Hershfield method of PMP estimation, the existing enveloping technique, and the proposed upgradation is discussed in the subsequent section.

## Method Details

### Hershfield Method of PMP estimation

Hershfield [25] proposed the basic equation for estimation of PMP, based on the general frequency equation suggested by Chow [31], as follows:

(1) $$X_{PMP}={\bar{X}}_{N}+K\times S_{N}$$

where, *X_PMP_*  is the PMP estimate for a particular location, ${\bar{X}}_{N}$is the mean of the AMDP series for *N* years at that location, *S_N_* is the standard deviation of the AMDP series at that location, *K* is the frequency factor for estimating PMP at that location, which can be determined from the following equation, proposed by [25],

(2) $$K=\frac{X_{m}-{\bar{X}}_{N-1}}{S_{N-1}}$$

where, *X_m_* is the maximum value in AMDP series of at the location, ${\bar{X}}_{N-1}$and $S_{N-1}$are the mean and standard deviation of the AMDP series, respectively for (*N*-1) years after removing the year with the maximum value.

Estimation of frequency factor *K* plays the key role in proper estimation of PMP. A very high value of *K* will lead to over-estimation of PMP and consequently an uneconomical design. In contrast, a lower value of *K* will lead to under-estimation of PMP, and therefore any structure designed based on that PMP estimate will be exposed to higher risk. So, the factor *K* has to be determined appropriately such that the design based on it, have a proper balance of economy and allowable risk. To do so, Hershfield [25] initially conducted a survey of 2700 stations (almost 90% stations from USA and remaining 10% from different other parts of world) to determine frequency factor *K* using Eqn. (2) and found *K* to vary within a range from slightly less than 3 to a highest value of 14.5. Hence, the highest value rounded to 15 was adopted as *K* for estimating 1 day-PMP using Eqn. (1). However, the universal applicability of *K* =15 was questioned by many researchers [32,33]. Even, Hershfield [1] himself found that *K* is not independent of rainfall magnitude and hence cannot have a universal value. To investigate further, the estimated *K* values (by Eqn. (2)) were plotted against corresponding mean AMDP (${\bar{X}}_{N}$) magnitude for all 2700 stations and observed that *K* has a general tendency to decrease with increase in ${\bar{X}}_{N}$ [1]. Hence, a single universal value of 15 may be too high as *K* for the areas with heavy rainfall and too low for arid areas. To overcome that, Hershfield [1] developed the upper envelope curve of *K* to determine the *K* value for a particular${\bar{X}}_{N}$, as given by the following equation,

(3) $$K=K_{A}e^{-a{\bar{X}}_{N}}$$

As per the Eq. (3), the envelope curve starts at some point *K_A_* for ${\bar{X}}_{N}=0$ and then exponentially decays as ${\bar{X}}_{N}$increases and becomes asymptotic towards the higher values of ${\bar{X}}_{N}$. So, in this technique, the frequency factor *K* no longer depends on the single highest value of *K* in the region, rather each grid point in the region has their own factor *K* as per the upper envelope curve and their mean AMDP magnitude (${\bar{X}}_{n}$). Hershfield [1] used 20 as the value of *K_A_* after analysing data from 2700 different locations as mentioned earlier, but this value may vary for different study areas. The slope of the exponentially decreasing curve depends on the factor ‘a’ (in Eqn. (3)), which is again a function of study area and duration of the rainfall.

### Proposed Upgradation in Enveloping Technique

#### Background

**Fig. 1:**
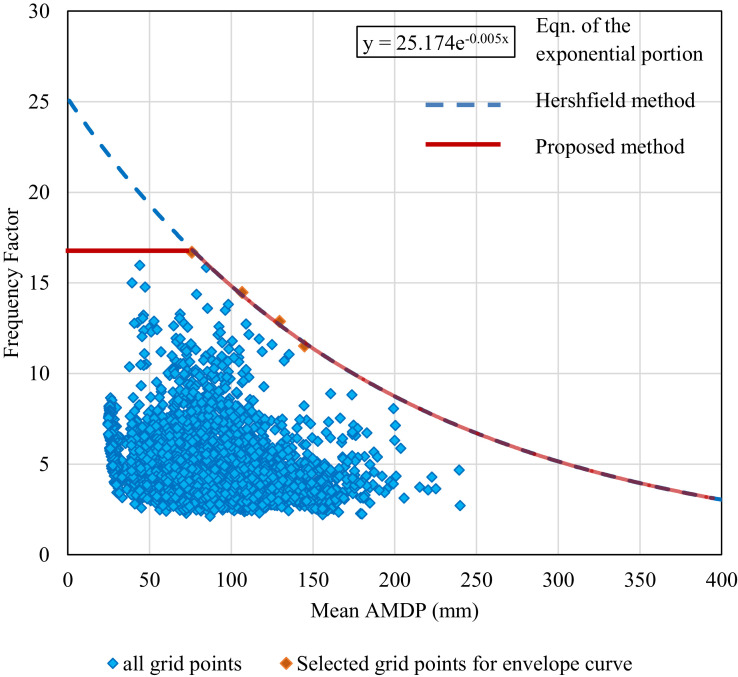
The scatter plot between mean AMDP and Frequency factor over India for 1901-2000 and the corresponding upper envelope curve according to Hershfield [1] and the proposed method in this study.

In the existing enveloping technique, it can be noticed that unusually high values may be assigned to the points with lower mean due to the steep nature of the exponential curve towards the left end. To understand this, the scatter plot between mean AMDP (${\bar{X}}_{N}$) and corresponding frequency factors (*K*), computed from daily gridded precipitation data across entire Indian mainland (4591 grid intersections) for the period 1901 to 2000 is shown in Fig. 1 (for more details about the data, please see the section *Comparison between Proposed and Existing Method* or [34]). From this scatter plot, a general trend of decreasing *K* with increasing ${\bar{X}}_{N}$ can be observed, with the *K* values mostly falling within a small range of 2-10. These observations match with the findings of other investigators [15,1,35,36]. The maximum value of *K* (say *K_m_*) can be found as 16.7, corresponding to ${\bar{X}}_{N}=75.9\;$mm from this scatter plot.

Now, some points on the possible upper envelop are selected (shown by orange diamond dots), and an exponential curve (blue dashed line) is fitted to those points, which designates the envelope curve following Hershfield [1]. This envelope curve meets the *K*-axis around 25 and thus provides excessive weightage (more than the maximum value i.e., 16.7) to the points with lower mean AMDP magnitude (less than 75.9 mm). This may not be a realistic reflection of the data points and hence leads to over-estimation of PMP over those low rainfall region. Hence, an upgraded technique of enveloping is introduced by Sarkar and Maity [17].

#### Proposed Technique

In the proposed technique, unlike a single envelope curve (as in existing Hershfield method), a composite envelope curve is considered. This composite curve consists of a straight line (parallel to x-axis) and an exponentially decay curve, same as the blue dashed line. The modified envelope curve is shown in Fig. 1 (red). The straight line and the exponential curve intersects at mean AMDP (${\bar{X}}_{N}$) $={\bar{X}}_{N}^{t}$ (t refers to transition point) and frequency factor (*K*) $=K_{m}$, where ${\bar{X}}_{N}^{t}$ denotes the mean AMDP at transition point and *K_m_*is the highest value of *K*. Thereby, the revised curve is expressed by Eqn. (4) as follows:

(4) $$K=\left\{ \begin{array}{cc} K_{m} & 0<{\bar{X}}_{N}\leq {\bar{X}}_{N}^{t}\\ K_{m}\;e^{-b\left({\bar{X}}_{N}-{\bar{X}}_{N}^{t}\right)} & {\bar{X}}_{N}>{\bar{X}}_{N}^{t} \end{array}\right.$$

The parameter ‘b’ determines the slope of the exponentially decreasing part of the envelope, and it is a function of the duration of interest and the study area.

It can also be noted that, as a special case, $K_{m}=K_{A}$ and ${\bar{X}}_{N}^{t}=0$ leads to the original Hershfield method, and in our opinion, it leads to erroneous estimation of PMP for the regions with relatively low rainfall magnitude. Thus, the revised envelope curve not only encapsulates all the observed frequency factors but also unusually high values are avoided for the low rainfall regions.

Referring to Fig. 1, the revised envelope curve consists of a straight line $K=K_{m}$ (=16.7, here) for the mean AMDP zero to ${\bar{X}}_{N}^{t}$ (= 75.9 mm, here) and an exponentially decaying curve for mean AMDP higher than 75.9 mm. Now, the new values of frequency factor can be read from this proposed envelope curve in order to determine the PMP at any location using Eqn. (1).

#### Comparison between Proposed and Existing Method

In order to compare the proposed method with the existing one, the Indian mainland is chosen as the study area which exhibits a wide spectrum of precipitation characteristics. Daily gridded observed rainfall from 4951 grid points, uniformly distributed throughout India at 0.25° latitude  ×  0.25° longitude resolution is procured from India Meteorological Department (IMD) for 110 years, i.e., from 1901-2010 [37]. Then, 1-day annual maximum rainfall values are extracted, and the series of AMDP values are prepared at each grid point. As an initial checking, it is noticed that at some grid points, especially in the north-east region, the recorded rainfall magnitude is zero throughout the year for many consecutive years. Such spurious cases are ignored from the analysis.

**Fig. 2:**
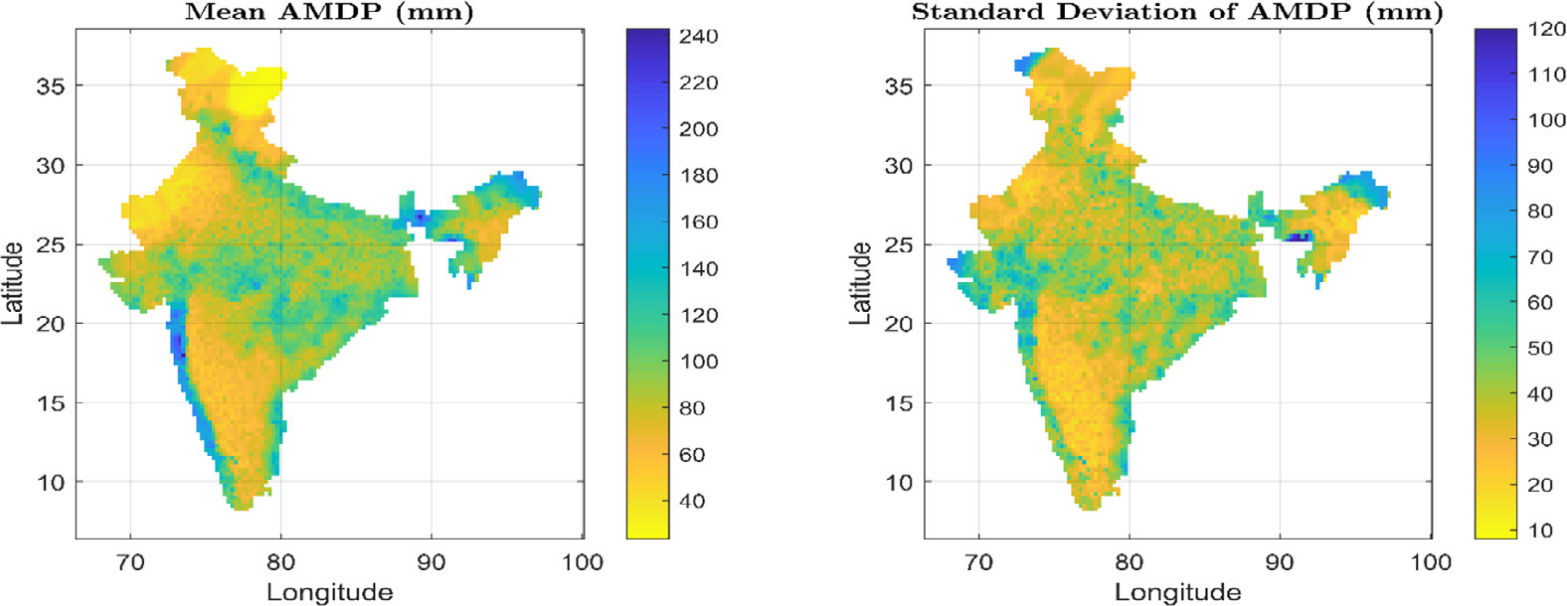
Mean and standard deviation of Annual Maximum Daily Precipitation (AMDP) over India for the last century (1901-2000).

The mean (${\bar{X}}_{N}$) and standard deviation (*S_N_*) of the AMDP series over the last century (1901-2000), at each grid point is evaluated and their spatial distribution is shown in Fig. 2, which depicts a wide range of extreme precipitation characteristics over India. From this figure, the dry regions (low AMDP) can be identified that are mostly confined in the North-West part of India (mostly Rajasthan), Southern peninsular India, Jammu-Kashmir portion, and some parts of North-East (Nagaland, Manipur, and East Assam). On the other hand, the wet regions with high AMDP are noticed in the western side of Western Ghats, East-central India, and Extreme North East portion (Arunachal Pradesh, Meghalaya, and West Assam). Then, the frequency factor (*K*) is estimated for each grid point using Eqn. (2), and the scatter plot of *K* vs. ${\bar{X}}_{N}$ is prepared and shown in Fig. 1. For this scatter plot, the upper envelope curves are developed as per Hershfield [1] and Sarkar and Maity [17], and are shown by blue dashed line and red solid line, respectively in Fig. 1. The equation of the exponential portion for both the envelope curves is shown in the inset of Fig. 1. Using these two envelope curves (or, Eqn. (4)), frequency factor is re-estimated depending on the mean AMDP magnitude (${\bar{X}}_{N}$) at a particular grid point.

**Fig. 3:**
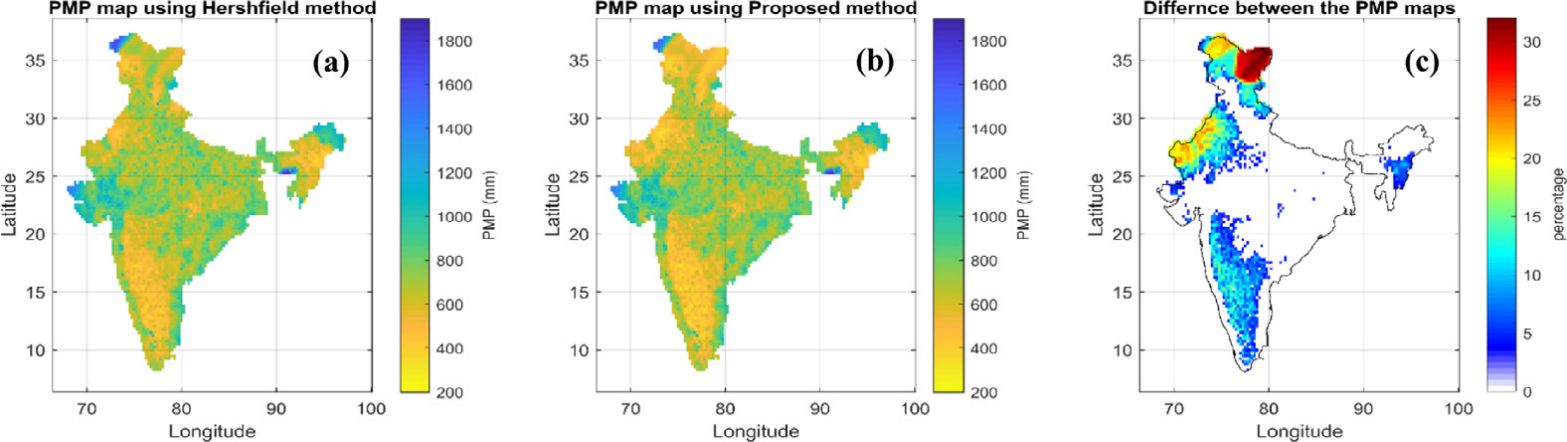
One-day PMP map developed for 1901-2000 (a) using Hershfield [1] method, (b) using the proposed method in this study, and (c) percentage difference between these two PMP estimates over India.

Now using ${\bar{X}}_{N}$,*S_N_* and this the re-estimated frequency factors in Eqn. (1), the PMP is determined for each grid point in India as per both the method, viz. Hershfield [1] and Sarkar and Maity [17]. The spatial distribution of the PMP, (or in other words, PMP maps) as per both the methods are shown in first two panels of Fig. 3 (Fig. 3a and 3b). Also, the percentage difference between both the PMP estimates is calculated according to the following equation and its spatial distribution is also shown in the third panel of Fig. 3 (Fig. 3c).

(5) $$D=\frac{PMP_{ext}-PMP_{pro}}{PMP_{pro}}\times 100\%$$

where, *PMP_ext_* is the PMP estimate as per the existing Hershfield method [1], *PMP_pro_* is the PMP estimate as per the proposed method i.e., Sarkar and Maity [17], and *D* is the difference between the estimates obtained from the existing and proposed method, expressed in percentage w.r.t. the proposed method. The spatial distribution of this percentage difference shows different extents of overestimation of PMP by Hershfield method, as compared to Sarkar and Maity [17], especially, for the dry regions with low rainfall extremes such as peninsular India, Rajasthan portion, Jammu-Kashmir etc. In some portions of Rajasthan, the extent of overestimation has gone up to 25%. Such observations indicate that the proposed upgradation in the enveloping technique provides more realistic spatio-temporal estimates of PMP, and thus avoids overestimation of PMP in case of the low extreme rainfall regions.

Further, the proposed method is applied to develop PMP maps over India for historical and future time periods to study the impact of climate change on PMP [17]. Readers can refer to [17] to understand the application of this method, and [34] to get access to the developed PMP data set and its future-projected changes over India.
